# The Complete Mitochondrial Genome of *Aleurocanthus camelliae*: Insights into Gene Arrangement and Genome Organization within the Family Aleyrodidae

**DOI:** 10.3390/ijms17111843

**Published:** 2016-11-07

**Authors:** Shi-Chun Chen, Xiao-Qing Wang, Pin-Wu Li, Xiang Hu, Jin-Jun Wang, Ping Peng

**Affiliations:** 1 Tea Research Institute, Chongqing Academy of Agricultural Science, Chongqing 402160, China; chensc0318@163.com (S.-C.C.); wangxiaoqing2891@126.com (X.-Q.W.); huxiang2016@126.com (X.H.); 2 Tea Science Department, College of Horticulture, Sichuan Agricultural University, Ya’an 611130, China; lipinwu@126.com; 3 Key Laboratory of Entomology and Pest Control Engineering, College of Plant Protection, Southwest University, Chongqing 400715, China; wangjinjun@swu.edu.cn

**Keywords:** *Aleurocanthus camelliae*, whitefly, mitochondrial genome, rearrangement, genome evolution

## Abstract

There are numerous gene rearrangements and transfer RNA gene absences existing in mitochondrial (mt) genomes of Aleyrodidae species. To understand how mt genomes evolved in the family Aleyrodidae, we have sequenced the complete mt genome of *Aleurocanthus camelliae* and comparatively analyzed all reported whitefly mt genomes. The mt genome of *A. camelliae* is 15,188 bp long, and consists of 13 protein-coding genes, two rRNA genes, 21 tRNA genes and a putative control region (GenBank: KU761949). The tRNA gene, *trnI*, has not been observed in this genome. The mt genome has a unique gene order and shares most gene boundaries with *Tetraleurodes acaciae*. Nineteen of 21 tRNA genes have the conventional cloverleaf shaped secondary structure and two (*trnS_1_* and *trnS_2_*) lack the dihydrouridine (DHU) arm. Using ARWEN and homologous sequence alignment, we have identified five tRNA genes and revised the annotation for three whitefly mt genomes. This result suggests that most absent genes exist in the genomes and have not been identified, due to be lack of technology and inference sequence. The phylogenetic relationships among 11 whiteflies and *Drosophila melanogaster* were inferred by maximum likelihood and Bayesian inference methods. *Aleurocanthus camelliae* and *T. acaciae* form a sister group, and all three *Bemisia tabaci* and two *Bemisia afer* strains gather together. These results are identical to the relationships inferred from gene order. We inferred that gene rearrangement plays an important role in the mt genome evolved from whiteflies.

## 1. Introduction

Over the last decade, the mitochondrial (mt) genome sequences have been frequently used to study species discrimination [1,2], molecule evolution [3,4,5,6], phylogenetic inferences [3,4,7,8] and population genetics [9,10], due to their small genome size, rapid evolutionary rate, low level or absence of sequence recombination and evolutionary conserved gene products [11,12]. With a few exceptions, animal mt genome is typically a circular double strand DNA molecule, with a size of 13–20 kb, consisting of a putative control region (CR) and 37 genes: 13 protein-coding genes (PCG), two ribosomal RNA genes (rRNA), and 22 transfer RNA genes (tRNA) [13,14,15,16,17]. The animal ancestral genome organization is retained in most insects, although minor changes were observed in some species [14,17]. In several groups of insects, mt genome organizations are not conservative and contain a lot of gene absences and rearrangements, pseudogenes or have been fragmented [5,8,18,19].

In recent years, whiteflies have become an almost worldwide problem for agriculturalists. Family Aleyrodidae, with around 1450 named species, belongs to the order Hemiptera and comprise a single superfamily, Aleyrodoidea, within the suborder Sternorrhyncha [20]. Recent studies showed that mt genome of whiteflies have numerous gene rearrangements and transfer RNA gene absences [21,22,23,24]. Transposition of gene block *cox3*-*trnG*-*nad3* and the position of *trnY* and *trnC* are typical in whitefly mt genomes. The number of absent genes ranges from one to five, and the mt genome of *Neomaskellia andropogonis* has lost the most tRNAs, e.g., *trnA*, *trnR*, *trnN*, *trnI* and *trnS_2_* [21]. The gene, *trnI*, is missed frequently and not found in the mt genomes of *Aleurochiton aceris*, *Tetraleurodes acaciae* and *N. andropogonis* [21].

The camellia spiny whitefly, *Aleurocanthus camelliae* Kanmiya and Kasai (Hemiptera: Aleyrodidae), is an important pest in tea plantations and poses a risk to tea quality and production. To understand how mt genomes evolved in the family Aleyrodidae, the complete sequence of the mt genome of *A. camelliae* was sequenced and comparative analyses of all reported whitefly mt genomes were conducted.

## 2. Results and Discussion

### 2.1. Genome Content and Nucleotide Composition

**Figure 1:**
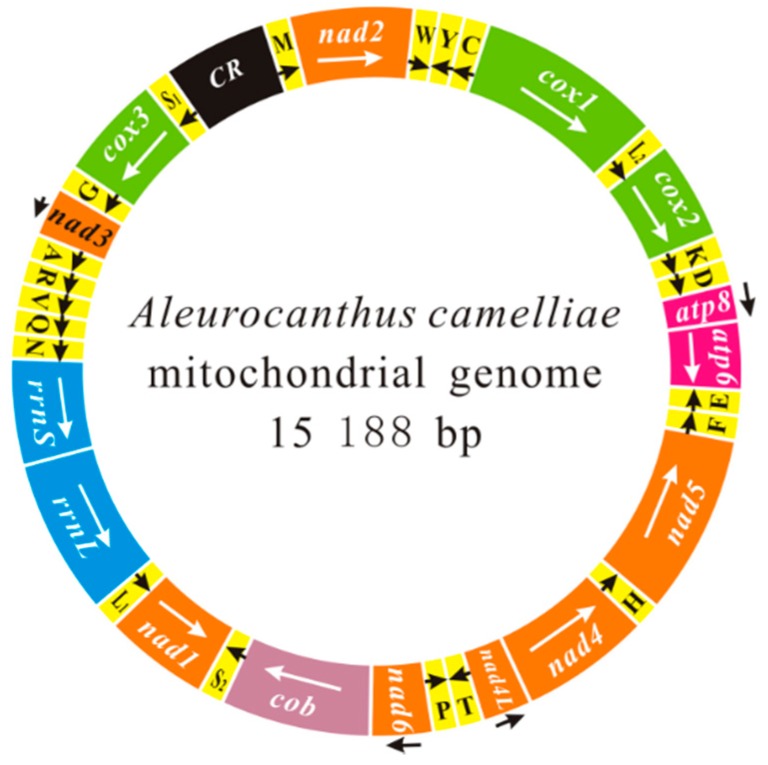
The mitochondrial genomes of *Aleurocanthus camelliae*. The transcriptional orientation is indicated with arrows. Protein-coding genes, ribosomal RNA genes and transfer RNA genes are shown in bright colors. All of them in the map follow standard abbreviations. tRNA genes for the two serine and two leucine tRNAs: S_1_ = AGN, S_2_ = UCN, L_1_ = CUN and L_2_ = UUR. The putative control region is indicated in black.

The mt genome of *A. camelliae* (GenBank accession KU761949) is a typical closed-circular and double stranded DNA molecule. The genome contains 36 of 37 genes usually found in animal mt genomes, including 13 protein-coding genes (PCG), two ribosomal RNAs, 21 transfer RNAs, and a putative control region (CR) (Figure 1 and Table 1). Genes are encoded by both strands of this mt genome: 14 genes on one strand whereas the rest on the other strand. The camellia spiny whitefly mt genome is in size of 15,188 bp, which is comparable to mt genome of other whiteflies ranging from 14,496 bp (*N. andropogonis*) to 18,414 bp (*Trialeurodes vaporariorum*) [21]. The major reason for the variation of genome size is the variable number of tandemly repeated elements in the putative control region. The control region of *A. camelliae* includes a poly-T sequence and two tandem repeat sequences, one repeat region consists of two 128 bp repeat units (13,423–13,678), and the other contains eight 19 bp repeat units (13,737–13,888). Similarly, the mt genome of *T. vaporariorum* has five 721 bp repeat units [21].

A total of 28 bp overlaps have been found at eight gene junctions of this mt genome, and the size of overlaps are ranging from 1 to 7 bp. Except for the putative control region, the genome has a total of 254 bp of intergenic sequence at 16 locations ranging from 1 to 94 bp, and the longest intergenic sequence is located between *rrnL* and *rrnS*. Unlike *A. camelliae*, a 53 bp long intergenic sequence locates between *rrnL* and *trnV* in the mt genome of *T. acaciae* [21].

As for all other insects, the nucleotide composition of the mt genome of *A. camelliae* is biased toward A and T nucleotides, with an A + T content of 70.90%. It consists of 69.49% in the protein coding genes, 76.61% in the ribosomal RNA genes, 77.19% in the transfer RNA genes and 66.86% in the putative control region, respectively (Table 2). Excluding the incomplete stop codons, a total of 3610 amino acids of protein-coding genes are encoded in the camellia spiny whitefly mt genome. The high A+T content is reflected in the codon usage, especially in the third position: codons for A or T (A = 33.88%, T = 40.46%) are strongly preferred over C or G (C = 14.19%, G = 11.47%) in the third codon position. The most frequent amino acids in the PCGs of the camellia spiny whitefly are Leucine (13.95%), Serine (10.37%), Phenylalanine (9.69%) and Isoleucine (9.36%) (Table 3).

The total length of all 13 protein-coding genes is 10,833 bp, which accounts for 71.33% of the whole length of the mt genome of *A. camelliae*. All PCGs initiate translation using an ATN codon (ATA for *atp8*, *nad2* and *nad4–6*; ATT for *atp6*, *cox2*, *nad1* and *nad3*; ATG for *cob*, *cox1*, *cox3* and *nad4L*). Three PCGs (*cox2*, *nad1* and *nad5*) have incomplete terminal codons consisting of “T-” nucleotide, and other stop with TAA and TAG (Table 1). Incomplete stop codons are common in animal mt genomes and could produce functional stop codons in polycistronic transcription cleavage and polyadenylation mechanisms [25,26].

**Figure 2:**
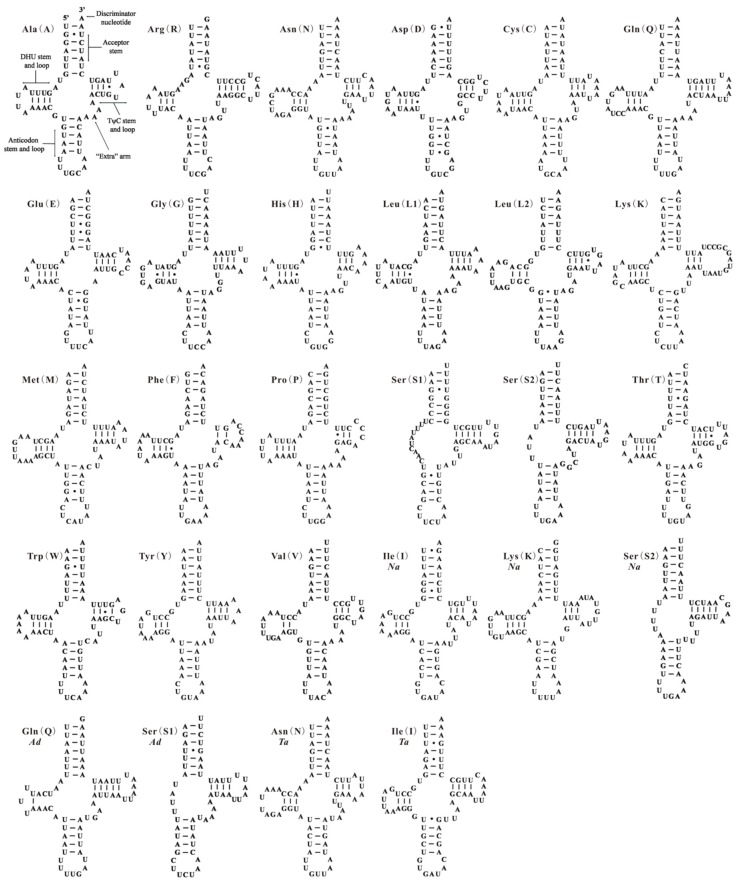
Putative secondary structures of the tRNA genes identified in this study. Bars indicate Watson-Crick base pairings, and dots between G and U pairs mark canonical base pairings appearing in RNA. Seven tRNA genes identified in this study of the mitochondrial genomes of *Aleurodicus dugesii* (*Ad*), *Neomaskellia andropogonis* (*Na*) and *Tetraleurodes acaciae* (*Ta*) with species name abbreviation below gene name.

All 22 tRNA genes usually found in the mt genomes of insects are present in *A. camelliae*, except for *trnI*. Seven of the 21 tRNA genes are encoded by one strand and the remaining genes are encoded by the other strand. The nucleotide length of tRNA genes are ranging from 56 bp (*trnS_2_*) to 71 bp (*trnQ*), and A + T content are ranging from 61.29% (*trnS_1_*) to 92.06% (*trnC*) (Table 1). In the mt genome of *A. camelliae*, 19 of 21 tRNA genes have the conventional cloverleaf shaped secondary structure and two (*trnS_1_* and *trnS_2_*) lacks the dihydrouridine (DHU) arm (Figure 2). The gene *trnS_1_* almost lacks the DHU arm in all metazoans [3,4,6], and *trnS_2_* without DHU arm also exists in *B. tabaci* Asia I [27] and *N. andropogonis* (Figure 2). In the 21 tRNA genes of *A. camelliae*, 39 unmatched base pairs were found, and they can be corrected through RNA-editing mechanisms that are well known for insect mtDNA [28].

### 2.2. Re-Annotation for Whitefly Mitochondrial Genomes

In the camellia whitefly mt genome, we identified 36 of 37 genes usually found in animal mt genomes, and gene *trnI* was not found. Gene absence is common in reported Aleyrodidae mt genomes, e.g., *A. aceris* without *trnI*, *Aleurodicus dugesii* without *trnS_1_* and *trnQ*, *T. acaciae* without *trnI*, *trnS_1_* and *trnN*, and *N. andropogonis* without *trnA*, *trnR*, *trnN*, *trnI* and *trnS_2_* [21]. The event of a gene disappearing has also occurred in mt genome of other animals, such as booklice [8,19] and arrow worm [29]. In some extreme cases, Cnidaria and Chaetognatha species lose nearly all their transfer RNA genes [29,30]. Current studies suggest that there are three reasons for gene absence of animal mt genomes. The first, the missing genes have been deleted and functionally replaced by nuclear tRNAs [31]. The second, the mt genome is fragmented and the missing genes are encoded by another unsequenced chromosome, like booklice [8,19], human lice [6], rotifer [32,33] and yellow tea thrips [18]. Thirdly, the disappeared genes actually exist in the circular DNA molecule, but have not been identified due to their rapid evolutionary rates. For example, earlier study indicated that the *nad3* gene was lost from the mt genome of *Metaseiulus occidentalis* [34], and the gene had been identified in a subsequent report [35].

**Figure 3:**
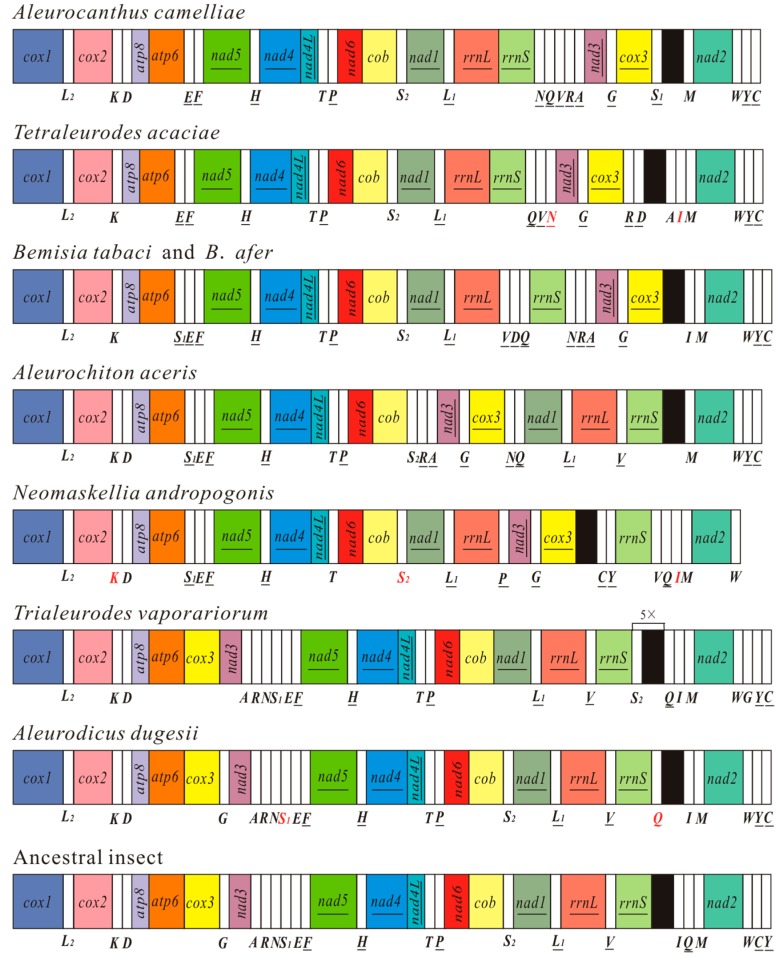
Arrangement of mitochondrial genes in the family Aleyrodidae and the hypothetical ancestral insect. Circular genomes have been arbitrarily linearized for ease of comparison. Gene names are the standard abbreviations used in the present study. Transfer RNA genes are designated by the single-letter amino acid codes, and red letters represent the tRNAs identified in this study. Genes which are underlined are encoded on the different strand from that of *cox1*. White boxes represent transfer RNA genes and bright colors represent 13 protein-coding genes and 2 ribosomal RNA genes.

In this study, we have constructed tRNA secondary structure using ARWEN additionally and aligned homologous gene sequences from other whiteflies. Five tRNA genes have been identified firstly in three whiteflies: *trnS_1_* and *trnQ* for *A. dugesii*, *trnI* and *trnN* for *T. acaciae* and *trnI* for *N. andropogonis* (Figure 2 and Figure 3, Table S1). For the mt genome of *N. andropogonis*, *trnK* and *trnS_2_* have been re-annotated and *cox2* gene has been reduced to 661 bp by tRNA punctuation model of *trnK* [36]. In original annotation, *cox2* gene (717 bp) is much longer than those of other Aleyrodidae species (661–667 bp), and *trnK* takes up the usual position of *trnS_2_*: thus, *trnS_2_* (14428–14493) huddles together with *trnW* (14430–14493). Gene *trnQ* of two whiteflies, *A. camelliae* and *A. dugesii*, have almost identical anticodon stem and loops, acceptor stems and TψC stem and loops. However, *trnQ* of *A. dugesii* nearly lost the DHU arm. The gene sequence has been much changed in the process of evolution, which increases the difficulty of gene identification. It infers that most gene absences of Aleyrodidae mt genome belong to the third situation of those mentioned above.

### 2.3. Gene Arrangement

The mt gene arrangement in *A. camelliae* is unique and different from that of the ancestral insect and other whiteflies (Figure 3). Mt gene rearrangement is also a common phenomenon for all Aleyrodidae species. As for most whiteflies, *A. camelliae* has the specific inverse transposition of gene block *cox3*-*trnG*-*nad3* and the position of *trnY* and *trnC* [21]. The gene order of camellia spiny whitefly is similar to that of *T. acaciae*. The two gene blocks, *atp8*-*atp6*-*trnE*-*trnF*-*nad5*-*trnH*-*nad4*-*nad4L*-*trnT*-*trnP*-*nad6*-*cob*-*trnS_2_*-*nad1*-*trnL_1_*-*rrnL*-*rrnS* and *trnQ*-*trnV*, only exist in the mt genomes of *A. camelliae* and *T. acaciae*. For the two whiteflies, gene *rrnS* is immediately followed by *rrnL*, with no transfer RNA gene but a long non coding sequence (94 and 53 bp) between them. However, the other whiteflies have one or more transfer genes between the two rRNAs [21,22,23,24,27].

There are numerous gene rearrangements have apparently occurred in the whitefly mt genomes, and the gene order map gives evidences to understand the process of rearrangements. For the major rearrangement, *cox3*-*trnG*-*nad3-trnA-trnR-trnN* firstly inverse transposed as a unit [21], subsequently, *trnA*, *trnR* and *trnN* have undergone transpositions independently. In Figure 3, *B. tabaci* and *B. afer* contain the whole inverse unit, *A. camelliae* and *A. aceris* with inverse *cox3-trnG-nad3-trnA-trnR*, and *T. acaciae* and *N. andropogonis* just have inverse *cox3-trnG-nad3*. Additionally, tRNA cluster *trnS_1_-trnE-trnF* has been left in the mt genomes of *B. tabaci*, *B. afer*, *A. aceris* and *N. andropogonis*. With *cox3*-*trnG*-*nad3-trnA-trnR-trnN* rearranged, the two ends of the unit became “hot spots” of tRNA gene transposition and insertion. For the mt genome of *A. camellia*, *trnQ-trnV* could be inserted after *cox3*-*trnG*-*nad3-trnA-trnR-trnN* rearranged, while six genes of the rearrangement unit maintain the original relative position. For *T. acaciae*, *A. aceris* and *N. andropogonis*, one or more tRNAs inserted into "hot spots" and *trnN* or all the three tRNAs (*trnA*, *trnR* and *trnN*) moved away. Thus, *trnA*, *trnR* and *trnN* in *N. andropogonis* have probably been rearranged several times and the sequences have been changed a lot. Once the tRNA genes lost their anticodons and functions, they would be eliminated. Rearrangement steps of *trnS_1_-trnE-trnF* are much clearer. The first step is the inversion of *trnS_1_* (*A. aceris* and *N. andropogonis*), the second is inversion of *trnE* (*B. tabaci* and *B. afer*) and the last is the transposition of *trnS_1_* (*A. camelliae* and *T. acaciae*). These rearrangement steps could be used to gain information of phylogenies.

To understand the mechanism that causes whitefly mt genome rearrangement, we conduct a comparative analysis among all Aleyrodidae mt genomes. Gene block *trnT*-*trnP* is common in all whitefly mt genomes, and a 71 bp non-coding region replaces *trnP* between *trnT* and *nad6* in *N. andropogonis* (Table S1). The mt genomes of *A. camelliae* and *T. acaciae* have the same situation, and a non-coding region instead of *trnV* locates between the two rRNA genes. These observations are highly consistent with the tandem duplication/random loss (TDRL) model, which is the widely accepted mechanism for local gene rearrangements in animal mt genomes [37]. However, most of genome rearrangements in Aleyrodidae are consistent with the intramolecular recombination mechanism, because gene inversions cannot be explained without some form of recombination [38,39,40]. The control region (CR) has been considered as a “hot spot” of recombination [41]. For whiteflies, gene block *cox3*-*trnG*-*nad3* usually translocates close to the control region. Besides *cox3*-*trnG*-*nad3*, many rearrangements with inversions occurred around CR, such as *rrnS*-*trnV* of *N. andropogonis*, *trnA* and *trnD* of *T. acaciae*, and *trnQ* of *A. dugesii*. The results indicate that both TDRL model and intramolecular recombination participate in genome rearrangement of whiteflies, and the latter plays a more important role.

### 2.4. Phylogenetic Analyses

**Figure 4:**
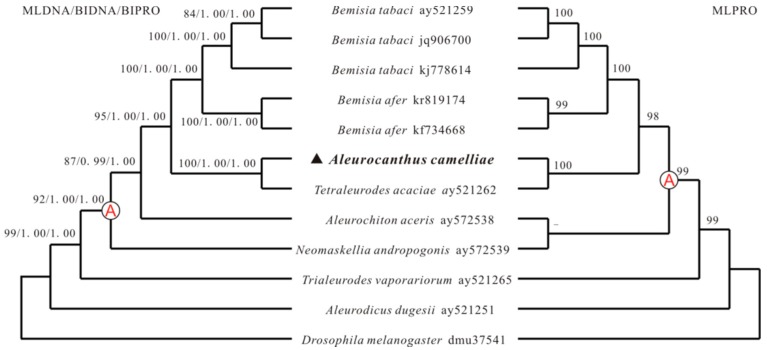
Phylogeny from Aleyrodidae mitochondrial genome sequences. Numbers above the branches show maximum likelihood (ML) bootstrap support values and Bayesian (BI) posterior probabilities for the phylogenies. Only support above 50% is shown. All mitochondrial genomes of clade A contain the rearrangement of *cox3*-*trnG*-*nad3*.

Two methods, maximum likelihood (ML) and Bayesian inference (BI), were used to determine phylogenetic relationships among 11 whiteflies from the family Aleyrodidae, with nucleotide and deducted amino acid sequences of mt genomes (Figure 4). Phylogenetic relationships among 11 whiteflies and *D. melanogaster* based on mt genome sequence are concordant with that inferred from mt gene order. *A. camelliae* and *T. acaciae* share most gene boundaries to each other and they are clustered into a branch of the phylogenetic tree with strong support (100% bootstrap value and 1.0 posterior probabilities). Similarly, three *B. tabaci* and two *B. afer* strains gather together and they have the same mitochondrial gene arrangement. In the cluster A, all species contain the insertion position of *cox3*-*trnG*-*nad3* and differ in tRNAs, that it is possible tRNA rearrangements followed the insertion of the transposed fragment in a common ancestor [21]. Based on rearrangement steps of *trnS_1_-trnE-trnF*, *A. aceris* and *N. andropogonis* split out firstly with the *trnS_1_* inversion, *B. tabaci* and *B. afer* followed them with *trnE* inversion. The gene order of the mt genome of *A. dugesii* and *T. vaporariorum* nearly have the ancestral gene composition and arrangement and contain a few tRNA rearrangements. Meanwhile, these two whiteflies locate more closely than the others to *D. melanogaster* in the phylogenetic tree, which insect has the same mt gene order as the ancestral insect [3,42]. It can be inferred that gene rearrangement plays an important role in the mt genome evolution in the family Aleyrodidae, and confirmed that the rearrangements are reliable phylogenetic markers [3,7,43].

## 3. Materials and Methods

### 3.1. Ethics Statement and Sample Collection

The camellia spiny whiteflies were collected at tea plantation in Yongchuan, Chongqing, China in 16 March 2015, and identified to species by morphology [2] and sequence of *cox1* [2,44] in 30 March 2015. Voucher specimens (#CQNKY-HE-02-01-01) were deposited at the Insect Collection, Tea Research Institute of Chongqing Academy of Agricultural Science, Chongqing, China. The sampling tea plantation belongs to Chongqing Academy of Agricultural Science, and the whitefly is a tea pest and not a protected species.

### 3.2. DNA Extraction and mt Genome Amplification

Total genomic DNA was extracted using a Dneasy Blood and Tissue Kit (Qiagen, Hilden, Germany) and stored at −20 °C. Referenced gene order of other whiteflies, the mt genome of *A. camelliae* were amplified in seven overlapping fragments by long-PCR with conserved insect primers [12,45,46] and specific primers designed in this study (Table 4). All fragments were sequenced and assembled into a contig with SeqMan (DNAStar).

Each long-PCR reaction is 25 μL in total volume, containing 1.0 μL each of forward primer (10 μM) and reverse primer (10 μM), 4.0 μL of dNTPs mix (each 2.5 mM), 1.0 μL of template DNA, 2.5 μL MgCl_2_ (25 mM), 2.5 μL of 10× LA PCR reaction buffer II, 12.75 μL ddH_2_O and 0.25 μL LA Taq DNA polymerase (5 U/μL, Takara, Dalian, China). All reactions were carried out using C1000™ thermal cyclers (Bio-RAD, Hercules, CA, USA) with the following conditions: 2 min denaturation at 94 °C, 35 cycles of 94 °C for 30 s, 55 °C for 30 s, 68 °C for 1–5 min (depending on target size, ~1 min/kb), followed by a final extension at 68 °C for 10 min. PCR products were directly sequenced with both forward and reverse PCR primers and internal primers by primer walking. All products in this study were sequenced by Life Technologies in Guangzhou, China.

### 3.3. Sequence Annotation and Analysis

The protein-coding genes were firstly identified by the ORF Finder (available online: http://www.ncbi.nlm.nih.gov/gorf/gorf.html) and rRNA genes by BLAST searches, then confirmed by alignment with homologous genes from other species of Aleyrodidae (Table 5). The transfer RNA genes were identified by cloverleaf secondary structure using ARWEN [47] with default parameters and tRNAscan-SE 1.21 [48] with the parameters: Search Mode = “EufindtRNA-Cove”, Genetic Code = “Invertebrate Mito” and Cove score cutoff = 1. The base composition was analyzed with BioEdit 7.2.5 (http://www.softpedia.com/get/Science-CAD/BioEdit.shtml). Sequences of mt genomes of other whiteflies were retrieved from GenBank (Table 5).

### 3.4. Phylogenetic Analyses

We indicated the relationship of the camellia spiny whitefly to seven other whiteflies (three strains of *Bemisia tabaci* and two strains of *Bemisi aafer*) with concatenated mt gene sequences of: (1) nucleotide sequences of 12 protein-coding genes and two rRNA genes (*atp8* excluded); (2) amino acid sequence of 12 protein-coding genes (*atp8* excluded). *Atp8* and tRNA genes were excluded because they are too short to align among 12 mt genomes. All sequences of genes were aligned individually by ClustalW in MEGA 5 [49]. Nucleotide sequences of PCGs were aligned at the amino acid level, then back-translated into nucleotide sequences. Poorly aligned sites of each alignment have been removed using the Gblocks server (available online: http://molevol.cmima.csic.es/castresana/Gblocks_server.html) [50] with all options for a stringent selection were chosen. We using DAMBE 5.3.9 to test substitution saturations of aligned nucleotide sequences [51]. Whole PCG nucleotide sequences would enter the next step if *I*_ss_ was significantly lower than *I*_ss.c_ (*p* < 0.05). Ten protein-coding genes passed this test, and the third codon positions of *nad4L* and *nad6* were excluded then the remainder passed the test. Subsequently, alignments of individual genes were concatenated. According to the Akaike Information Criterion, the best fit models for nucleotide sequence alignment was determined by jModelTest 2.1.4 [52,53], and amino acid sequence alignment by ProtTest 3.2 [54]. Then, GTR+I+G model and MtArt+I+G model were chosen. The two concatenated alignments were used in maximum likelihood (ML) and Bayesian inference (BI) with PhyML3.0 (available online: http://www.atgc-montpellier.fr/phyml/) [55] and MrBayes v3.2.2 [56,57]. ML analyses were performed with substitution models “GTR” or “MtArt”, type of tree improvement “SPR & NNI”, and the shape parameters and the propotions of invariable sites were estimated by jModelTest 2.1.4 and ProtTest 3.2. For BI analyses, four independent Markov chains were simultaneously run for 2,000,000 generations with a heating scheme (temp = 0.2); Trees were sampled every 100 generations (sample-freq = 100) and the first 25% were discarded as burn-in. Stationarity was considered to be reached when the average standard deviation of split frequencies was below 0.01 [58].

## 4. Conclusions

We sequenced the complete mt genome of camellia spiny whitefly, *Aleurocanthus camelliae*. This mt genome shares many features with those reported insect mt genomes. It is a typical closed-circular and double stranded DNA molecule in size of 15,188 bp. Distinctively, it has unique gene arrangement and *trnI* could not be identified. The mt genome encodes 13 protein-coding genes, 2 ribosomal and 22 transfer RNA genes, but no *trnI*. Gene absence is a common phenomenon of whitefly mt genomes. Using ARWEN and homologous sequence alignment, we have identified five tRNA genes and revised genome annotations of *A. dugesii*, *N. andropogonis* and *T. acaciae* in this study. The gene order of camellia spiny whitefly is similar to that of *T. acaciae*. Phylogenetic relationships among 11 whiteflies and *D. melanogaster* based on mt genome sequence are concordant with that inferred from mt gene order. This infers that gene rearrangement plays an important role in the mt genome evolved from whiteflies.

## Figures and Tables

**Table 1:** Annotation and gene organization of the mitochondrial genome of *Aleurocanthus camelliae*.

Gene ^a^	Region	Size	Inc ^b^	AT%	AT-Skew ^c^	GC-Skew	Start Codon	Stop Codon	Anticodon
*cox1*	1–1539	1539	1	64.39	−0.322	0.263	ATG	TAA	-
*trnL_2_*	1542–1606	65	2	70.77	−0.087	0.474	-	-	TAA
*cox2*	1607–2270	664	0	68.67	−0.197	0.202	ATT	T- ^e^	-
*trnK*	2271–2340	70	0	71.43	0.000	−0.100	-	-	CTT
*trnD*	2347–2408	62	6	66.13	−0.171	0.333	-	-	GTC
*atp8*	2418–2564	147	9	62.59	−0.217	0.273	ATA	TAA	-
*atp6*	2558–3208	651	−7	71.27	−0.388	0.241	ATT	TAG	-
*trnE*	3233–3293	61	24	72.13	0.000	0.059	-	-	TTC
*trnF*	3301–3363	63	7	76.19	0.292	0.067	-	-	GAA
*nad5*	3364–5023	1660	0	73.13	0.137	−0.166	ATA	T-	-
*trnH*	5024–5084	61	0	81.97	0.040	0.455	-	-	GTG
*nad4*	5086–6366	1281	1	69.95	−0.007	−0.179	ATA	TAG	-
*nad4L*	6363–6659	297	−4	76.77	−0.018	−0.246	ATG	TAA	-
*trnT*	6661–6724	64	1	76.56	−0.102	0.333	-	-	TGT
*trnP*	6723–6784	62	−2	72.58	0.111	−0.059	-	-	TGG
*nad6*	6816–7250	435	31	70.57	−0.270	0.328	ATA	TAA	-
*cob*	7250–8383	1134	−1	64.90	−0.351	0.186	ATG	TAA	-
*trnS_2_*	8384–8439	56	0	80.36	−0.067	0.273	-	-	TGA
*nad1*	8450–9359	910	10	72.86	0.005	−0.198	ATT	T-	-
*trnL_1_*	9360–9422	63	0	84.13	0.132	0.200	-	-	TAG
*rrnL*	9423–10612	1190	0	77.23	0.134	−0.055	-	-	-
*rrnS*	10707–11435	729	94	75.99	0.022	0.074	-	-	-
*trnN*	11483–11548	66	47	78.79	0.115	0.143	-	-	ATT
*trnQ*	11553–11623	71	4	87.32	0.000	−0.111	-	-	TTG
*trnV*	11617–11680	64	−7	73.44	−0.021	0.059	-	-	TAC
*trnR*	11678–11742	65	−3	73.85	−0.042	0.059	-	-	TCG
*trnA*	11744–11805	62	1	75.81	−0.021	0.200	-	-	TGC
*nad3*	11806–12159	354	0	72.60	−0.276	0.052	ATT	TAA	-
*trnG*	12161–12222	63	1	82.54	−0.038	0.273	-	-	TCC
*cox3*	12223–13009	786	0	66.92	−0.198	0.115	ATG	TAA	-
*trnS_1_*	13025–13086	62	15	61.29	−0.263	0.083	-	-	TCT
CR ^d^	13087–13961	875	0	66.86	−0.050	0.117	-	-	-
*trnM*	13962–14028	67	0	77.61	0.077	−0.067	-	-	CAT
*nad2*	14029–15003	975	0	68.72	−0.287	0.279	ATA	TAA	-
*trnW*	15002–15064	63	−2	82.54	0.077	0.091	-	-	TCA
*trnY*	15063–15124	60	−2	86.67	0.154	0.500	-	-	GTA
*trnC*	15125–15187	63	0	92.06	0.138	0.200	-	-	GCA

^a^ Genes located in the different strand from that of *cox1* are underlined; ^b^ inc = intergenic nucleotides, indicating gap nucleotides (positive value) and overlapping nucleotides (negative value) of two adjacent genes; ^c^ AT-skew = (A − T)/(A + T), GC-skew = (G − C)/(G + C); ^d^ CR = control region (putative); ^e^ “T-” indicates the protein-coding gene terminate translation with single nucleotide “T”.

**Table 2:** Nucleotide composition of the mitochondrial genome of *Aleurocanthus camelliae*.

Region	A%	C%	G%	T%	AT%	AT-Skew	GC-Skew
Full length	31.02	12.38	16.72	39.87	70.90	−0.125	0.149
PCGs	29.27	14.12	16.64	39.96	69.49	−0.154	0.082
1st codon	35.12	13.20	20.62	31.05	66.37	0.061	0.219
2nd codon	18.81	17.70	15.12	48.37	68.08	−0.440	−0.078
3rd codon	33.88	11.47	14.18	40.47	74.02	−0.089	0.106
tRNA genes	39.51	9.75	13.04	37.71	77.19	0.023	0.145
rRNA genes	41.90	11.67	11.57	34.86	76.61	0.092	−0.004

**Table 3:** Codon usage for the 13 mitochondrial protein-coding genes of *Aleurocanthus camelliae*.

Codon	N	%	RSCU	Codon	N	%	RSCU	Codon	N	%	RSCU
UAA (*)	8	0.22	1.60	AAA (K)	79	2.19	1.30	AGA (S)	76	2.11	1.63
UAG (*)	2	0.06	0.40	AAG (K)	43	1.19	0.71	AGC (S)	23	0.64	0.49
GCA (A)	62	1.72	1.56	CUA (L)	91	2.52	1.54	AGG (S)	22	0.61	0.47
GCC (A)	23	0.64	0.58	CUC (L)	16	0.44	0.27	AGU (S)	55	1.52	1.18
GCG (A)	14	0.39	0.35	CUG (L)	42	1.16	0.71	UCA (S)	83	2.30	1.78
GCU (A)	60	1.66	1.51	CUU (L)	87	2.41	1.48	UCC (S)	18	0.50	0.39
UGC (C)	17	0.47	0.74	UUA (L)	178	4.93	1.33	UCG (S)	17	0.47	0.36
UGU (C)	29	0.80	1.26	UUG (L)	90	2.49	0.67	UCU (S)	80	2.22	1.71
GAC (D)	22	0.61	0.70	AUA (M)	225	6.23	1.54	ACA (T)	76	2.11	1.62
GAU (D)	41	1.14	1.30	AUG (M)	67	1.86	0.46	ACC (T)	19	0.53	0.40
GAA (E)	57	1.58	1.37	AAC (N)	48	1.33	0.63	ACG (T)	6	0.17	0.13
GAG (E)	26	0.72	0.63	AAU (N)	104	2.88	1.37	ACU (T)	87	2.41	1.85
UUC (F)	46	1.27	0.26	CCA (P)	26	0.72	1.11	GUA (V)	85	2.35	1.30
UUU (F)	304	8.42	1.74	CCC (P)	18	0.50	0.77	GUC (V)	21	0.58	0.32
GGA (G)	62	1.72	1.39	CCG (P)	13	0.36	0.55	GUG (V)	49	1.36	0.75
GGC (G)	20	0.55	0.45	CCU (P)	37	1.02	1.57	GUU (V)	106	2.94	1.63
GGG (G)	55	1.52	1.23	CAA (Q)	29	0.80	1.14	UGA (W)	68	1.88	1.36
GGU (G)	42	1.16	0.94	CAG (Q)	22	0.61	0.86	UGG (W)	32	0.89	0.64
CAC (H)	22	0.61	0.86	CGA (R)	18	0.50	1.60	UAC (Y)	48	1.33	0.65
CAU (H)	29	0.80	1.14	CGC (R)	5	0.14	0.44	UAU (Y)	99	2.74	1.35
AUC (I)	48	1.33	0.28	CGG (R)	12	0.33	1.07	-	-	-	-
AUU (I)	291	8.06	1.72	CGU (R)	10	0.28	0.89	-	-	-	-

* represents stop codon.

**Table 4:** PCR primers for amplification of the mitochondrial genome of *Aleurocanthus camelliae*.

Region	Primer Name	Primer Sequence (5′–3′)	References
*cox1-nad5*	C1-J-2195	TTGATTTTTTGGTCATCCAGAAGT	[45]
	N5-N7793	TTAGGTTGRGATGGNYTAGG	[12]
*nad5-cob*	AC-N5J	CAGCAGTTACTAGAGTTGATGAGT	In this study
	AC-CBN	TCTCCACAAACAATAAAACAAGC	In this study
*cob-rrnL*	CBF1	TATGTACTACCATGAGGACAAATATC	[46]
	AC-16SN	GTCTAAGTCTTAAATAAACTCTGC	In this study
*rrnL*	16Sar	CGCCTGTTTAACAAAAACAT	[46]
	16Sbr	CCGGTCTGAACTCAGATCACGT	[46]
*rrnL-rrnS*	AC-16SJ	TTAGAGGAACCTGCTTAGTAATC	In this study
	AC-12SN	GCAGAGTTTATTTAAGACTTAGAC	In this study
*rrnS*	12SRNA-F	AAACTAGGATTAGATACCCTATTAT	[46]
	12SRNA-R	AAGAGCGACGGGCGATGTGT	[46]
*rrnS-cox1*	AC-12SJ	CCCTGATACAAAAGGTACAAGTCT	In this study
	AC-C1N	CCCATAATAGCAAATACAATCCCT	In this study

**Table 5:** GenBank accession numbers and mitochondrial genome sizes of the species included in phylogenetic analysis in this study.

Species	Genome Size	Acc. Number	References
*Bemisia tabaci* complex sp. Asia I	15,210 bp	KJ778614	[27]
*Bemisia tabaci*	15,632 bp	JQ906700	[23]
*Bemisia tabaci*	15,322 bp	AY521259	[21]
*Bemisia afer*	15,300 bp	KR819174	[24]
*Bemisia afer*	14,968 bp	KF734668	[22]
*Tetraleurodes acaciae*	15,080 bp	AY521262	[21]
*Neomaskellia andropogonis*	14,496 bp	AY572539	[21]
*Aleurochiton aceris*	15,388 bp	AY572538	[21]
*Aleurodicus dugesii*	15,723 bp	AY521251	[21]
*Trialeurodes vaporariorum*	18,414 bp	AY521265	[21]
*Drosophila melanogaster*	19,517 bp	DMU37541	[42]

## Supplementary Materials

Supplementary materials can be found at www.mdpi.com/1422-0067/17/11/1843/s1.
